# The Use of Psychotherapy for Refractory Irritable Bowel Syndrome: A Systematic Review

**DOI:** 10.7759/cureus.54138

**Published:** 2024-02-13

**Authors:** Ethan Slouha, Ahmed Mohamed, Bansari Patel, Ziyad Razeq, Lucy A Clunes, Theofanis F Kollias

**Affiliations:** 1 Anatomical Sciences, St. George's University School of Medicine, St. George's, GRD; 2 Pharmacology, St. George's University School of Medicine, St. George's, GRD; 3 Medicine, St. George's University School of Medicine, St. George's, GRD; 4 Microbiology, Immunology and Pharmacology, St. George's University School of Medicine, St. George's, GRD

**Keywords:** gut-directed hypnotherapy, refractory irritable bowel syndrome, cognitive behavior therapy, psychotherapy, irritable bowl syndrome

## Abstract

Irritable bowel syndrome (IBS) is a common yet debilitating and chronic condition that consists of disturbances in bowel habits and abdominal pain that is frequently relieved with defecation. While the first line of treatment for IBS is pharmacological treatment, this has been shown to fail, leading to the patient being classified as having refractory IBS. The quality of life (QOL) of these patients is greatly hindered; in this case, there are rarely moments of relief. Additional modalities of treatment have been employed in classical cases of IBS, such as psychotherapy, and research has started to evaluate its effectiveness with refractory IBS. Both cognitive behavioral therapy (CBT) and gut-directed hypnotherapy (GDH) are effective in treating classical IBS as they restructure and bring a state of meditation to the patient, allowing them to work through the symptoms. The question is whether it remains successful in refractory cases. This systematic review was conducted with strict adherence to PRISMA guidelines with an initial inquiry resulting in 28,978 publications through PubMed, ScienceDirect, and ProQuest databases. Through automatic and manual screening processes, articles that were peer-reviewed experimental or observation publications done between 2003 and 2023 were included in this study, resulting in 21 publications. Across all studies evaluating CBT, it was consistently found to be successful in improving symptom severity and frequency, QOL, and extracolonic symptoms such as anxiety and depression. When broken down into delivery methods, minimal contact CBT was found to be just as, if not superior, to standard contact. Within this, telephone-delivered CBT was superior to web-delivered CBT. GDH and biofeedback therapy were found to also significantly improve all domains of IBS with no difference between them. Acceptance and commitment therapy were found only to improve associated symptoms. However, there was no significant improvement in their QOL, whereas integrative group therapy found no significant improvement in any domain. Because IBS is so common and crippling to those affected, its crucial to continuously improve QOL through advancement in treatment modalities. Further research should focus more on other modes of therapy as success has been shown in standard therapeutic techniques.

## Introduction and background

Irritable bowel syndrome (IBS) is a very common gastrointestinal condition affecting up to 12% of individuals in the United States that presents as chronic abdominal pain and disturbances in bowel habits, significantly impacting the patient’s quality of life (QOL) [1]. IBS is classified via the Rome Criteria, which include symptoms longer than six months, symptom activity during the last three months, symptom frequency at least one day per week, abdominal pain, and symptoms related to defecation with stool consistency change [1,2]. The mechanism of IBS is relatively unknown, with proposed theories relating to inflammation, dysmotility, altered intestinal microbiota, and visceral hypersensitivity [1,2]. The basics of treatment involve a strong patient-physician relationship, as this is a lifelong disease process. Extensive education and plenty of reassurance by the provider must always occur in the first few assessments [1,2]. Following this, the first line of treatment is pharmaceutical therapies to treat the symptoms [1,2]. Treatment varies depending on the symptom experienced; for example, constipation is treated with polyethylene glycol, psyllium, or chloride channel activators, while diarrhea is treated with opioid agonists, antibiotics, probiotics, and 5-HT3 antagonists [1,2]. Treatment for abdominal pain includes selective serotonin reuptake inhibitors, tricyclic antidepressants, antispasmodics, and even peppermint oil [1,2].

In many instances, pharmaceutical therapy fails to provide relief, and the patient is said to have refractory IBS [3]. While the diagnosis of refractory is subjective, ultimately, the patient finds minimal to no relief from their IBS symptoms [3]. In instances of traditional IBS, psychotherapy has been implemented to aid in maintaining IBS [1,2]. Common therapies employed include cognitive behavioral therapy (CBT) and gut-directed hypnotherapy (GDH), both shown to be successful [1,2]. CBT, in practice, halts patients from performing automatic thoughts, restructures cognitive distortions, and improves the patients underlying beliefs to improve the outcome of their thought process [4]. GDH teaches patients to employ a state of calmness during symptoms and essentially mentally relaxes the tension caused by the pain to create much-needed relief [5]. Studies have employed these methods, as well as other psychotherapeutic techniques, in patients with refractory pain and evaluated their success.

Aim

Since IBS is common and significantly debilitating, it is crucial to address varying treatment modalities to further aid in management. The diagnosis is more critical when refractory to other treatment techniques, such as pharmaceutical interventions. This paper aims to evaluate the application of psychotherapy in addition to medical management in refractory IBS patients. Publications on CBT, GDH, and other articles covering up-and-coming therapeutic approaches were pooled. All articles assessed the impact of therapy on symptoms severity, frequency, QOL, and extracolonic symptoms such as anxiety and depression. The aim is to present data to reinforce the need for additional treatment modalities, especially ones with limited adverse effects.

## Review

Methods

For this systematic review, the methods for searching and analyzing the articles strictly adhered to the PRISMA guidelines presented by Liberati et al. [6]. The three databases used to conduct this query were PubMed, ScienceDirect, and ProQuest and were tailored to focus on the years between January 1, 2013, and December 10, 2023. The keywords were used to focus the search: ‘Psychotherapy and refractory irritable bowel syndrome’, ‘Cognitive behavioral therapy and refractory irritable bowel syndrome,’ and ‘Hypnotherapy and refractory irritable bowel syndrome’. This review focused on peer-reviewed experimental and observational publications, with an initial search resulting in 28,978 publications. Publications excluded were done due to being either duplications, published before 2013, or not written in English. Following the exclusion process, the lasting publications were manually evaluated by four independent co-authors regarding their title, study type, abstract, and full-text availability. The conclusion of the screening process included an in-depth analysis of the text to ensure it correlated with this paper’s aim. A total of 21 publications were attained via the following criteria.

Inclusion Criteria

Selected publications following the screening process were included based on meeting the following criteria: studies performed on humans, those with full-text availability, peer-reviewed experimental and observational studies, those that covered only refractory IBS, and those published between 2013 and 2023.

Exclusion Criteria

**Figure 1:**
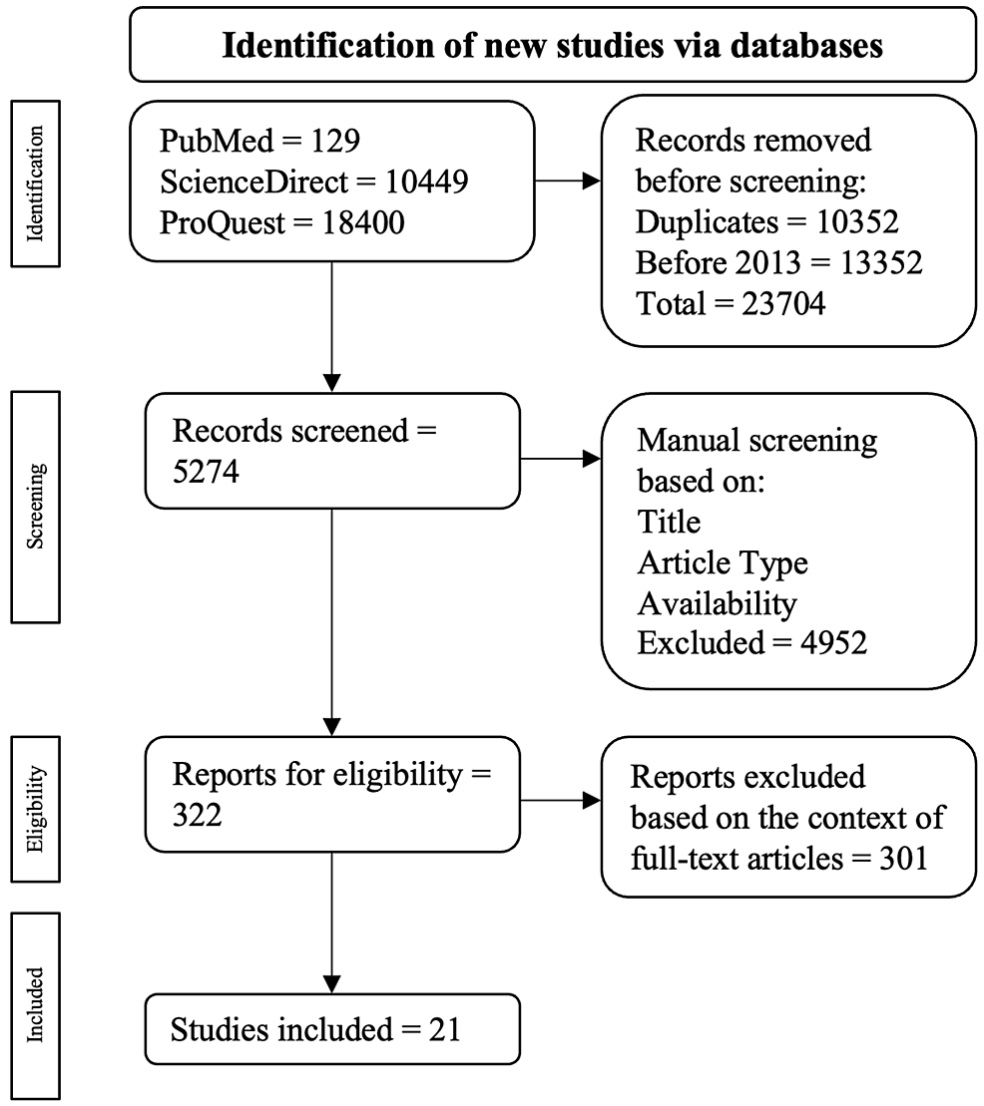
Algorithm employed using based on the inclusion and exclusion criteria. The flowchart was adapted to PRISMA guidelines [6].

Excluded publications were done for one of the following criteria: published in a language other than English, published before 2003, and/or duplicates acquired during the search. The implementation of the screening process is displayed in Figure 1.

Bias

All studies included thorough explanations of their procedures and methods; however, the use of secondary analysis in a couple of studies has the potential to impact the paper's bias as a whole. The application of this secondary analysis is through the use of only new information and not a duplication of the same result; it is comfortable to state an overall moderate bias using the GRADE (grading of recommendation, development, and evaluation) scale. The GRADE scale requires individual assessments of publications to evaluate imprecision and publication type that can construe bias.

Results

A total of 28,978 publications were initially found: 129 were from PubMed, 10,449 were from ScienceDirect, and 18,400 were from ProQuest. Among the exclusions, 10,352 were duplicate publications, and 13,352 were published before 2013. This led to 23,704 publications being excluded at the end of the first part of the systematic process, leaving 5,274 publications to be assessed. Publications were then manually assessed by four independent co-authors based on their full-text availability, title, and study type, leaving 322 publications for full-text assessment. Ultimately, 21 publications were selected.

Evaluation of CBT for the management of IBS had one of the most amount of research done with all studies indicating significant success in improving IBS-Symptom Severity Score (IBS-SSS), frequency of symptoms, QOL, and anxiety and depression. Several studies compared Standard Contact-CBT (S-CBT) and Minimal Contact-CBT (MC-CBT) and observed that MC-CBT was just as efficacious as S-CBT, even more so in all domains except anxiety. Within MC-CBT, telephone-delivered CBT (TCBT) and web-delivered CBT (WCBT) were compared, and it was found that while both stayed consistent with the typical results of CBT for IBS, TCBT had great improvement in all domains, but was not as cost-effective as WCBT. Hypnotherapy was also found to significantly improve symptoms of refractory IBS as well in all domains, along with biofeedback therapy; there were no differences between these techniques. Two additional studies focused on new therapeutic modalities: acceptance and commitment therapy (ACT) and integrative group therapy. ACT was found to enhance IBS-SSS, GI-specific anxiety, and acceptance of IBS; however, it did not improve the QOL of patients. While encompassing multiple modalities, integrative group therapy did not yield significant improvements throughout the domains. Table 1 depicts the summary analysis of literature used in this review.

**Table 1:** Summary of the literature used in this review per PRISMA guidelines [6]. MC-CBT - Minimal-Contact Cognitive Behavioral Therapy; IBS - Irritable Bowel Syndrome; S-CBT - Standard-Contact Cognitive Behavioral Therapy; CBT - Cognitive Behavioral Therapy; QOL - Quality of Life; GI - Gastrointestinal; ACT - Acceptance and Commitment Therapy; IBS-SSS - IBS Symptom Severity Scale; GDH - Gut-Directed Hypnotherapy; TCBT - Telephone-Cognitive Behavioral Therapy; WCBT - Internet-Cognitive Behavioral Therapy; TAU - Treatment As Usual

	Author	Country	Design & Study Population	Findings	Conclusion
1	Hughes et al., 2020 [7]	UK	Randomized Control Trial (n = 34)	The findings revealed that participants generally viewed CBT as a credible approach for managing IBS. Participants reported positive changes in their understanding of IBS, attitudes toward the condition, ability to recognize patterns related to IBS, and IBS-related behaviors. In line with the results of the larger trial, participants noted lasting positive effects on their IBS symptoms and social lives.	In conclusion, both TCBT and WCBT interventions for IBS were well-received by participants and had enduring positive impacts on their understanding of IBS, IBS-related behaviors, symptoms, and overall QOL. These findings suggest that these forms of CBT could expand access to effective IBS treatment.
2	Kikuchi et al., 2022 [8]	Japan	Experimental Study (n = 114)	The group that received guided CBT-based interoceptive exposure showed significantly more positive responses or improvements regarding two primary outcomes than those on the waiting list.	Guided CBT-based interoceptive exposure showed positive responses or improvements in treating refractory IBS.
3	Lackner et al., 2022 [9]	USA	Experimental Study (n = 130)	For CBT patients, a clinical improvement in cognitive flexibility was associated with changes in symptom severity, abdominal pain, and quality of life as opposed to those with nonspecific education. Neither showed changes in psychological flexibility or use of emotion regulation strategies.	Using CBT showed an improvement in cognitive flexibility, symptom severity, abdominal pain, and quality of life.
4	Lackner et al., 2019 [10]	USA	Secondary Analysis (n = 436)	The study revealed significant results concerning IBS symptom improvement. Patients who underwent CBT exhibited higher rates of symptom improvement compared to those who received education. This difference was particularly notable among individuals with low levels of trait anxiety or anxiety sensitivity.	The examination of individuals with IBS resistant to conventional treatments revealed an association between initial levels of trait anxiety and anxiety sensitivity and enhanced gastrointestinal symptom improvement after undergoing CBT in contrast to IBS education.
5	Lackner et al., 2021 [11]	USA	Randomized Control Trial (n = 436)	A positive treatment expectancy, self-efficacy, and patient-therapist agreement were achieved for CBT in early and post-treatment. CBT had comparable rapid response rates of 43-45%, remarkably greater than education's rapid response rate of 22%.	Mediation of CBT-induced symptom improvement may be due to CBT-specific self-efficacy and nonspecific treatment expectancy and task agreement that influence each other to improve, catalyze, and sustain gastrointestinal symptom relief.
6	Lackner et al., 2018 [12]	USA	Experimental Study (n = 436)	MC-CBT showed superiority over the education condition regarding treatment responders starting at 2 weeks. Differences in patient-reported outcomes lost significance at 6 months, and patient satisfaction was higher for CBT conditions than for education conditions. The study also confirmed the non-inferiority of MC-CBT compared to S-CBT.	Patients with IBS responded better to MC-CBT compared to those who only received education about the condition and its course. Also, MC-CBT was just as effective as S-CBT in improving outcomes for patients with IBS.
7	Lackner et al., 2019 [13]	USA	Secondary Analysis (n = 436)	MC-CBT and S-CBT maintained positive treatment responses at every follow-up at 39 and 33%, respectively, whereas education therapy maintained 19% of subjects, which was significantly lower. IBS-SSS improved over time.	Clinic and home-based CBT yielded relief in symptoms that extended to 12 months post-treatment in treatment-refractory IBS.
8	McCrone et al., 2021 [14]	UK	Experimental Study (n = 558)	TCBT costs more than treatment as usual and adds more quality-adjusted life years. WCBT costs more than treatment as usual and has more quality-adjusted life years. With informal care and lost employment, TCBT was more expensive than treatment as usual, and WCBT cost less than treatment as usual.	TCBT and WCBT had more quality-adjusted life years and higher costs than treatment as usual. Both are cost-effective from a healthcare viewpoint. Adding missing data decreases cost effectiveness except for WCBT.
9	Everitt et al., 2019 [15]	UK	Randomized Control Trial (n = 323)	The TCBT group and WCBT group exhibited a mean IBS-SSS score of 40.5 and 12.9 points lower than the TAU group, respectively. The mean WSAS score decreased by 3.1 and 1.9 points in the TCBT and WCBT groups compared to the TAU groups.	During the 24-month follow-up, both CBT groups demonstrated sustained improvements in IBS compared to the TAU group. However, it's worth noting that some of the previous gains observed at the 12-month mark were somewhat reduced.
10	Everitt et al., 2019 [16]	UK	Experimental study (n = 558)	IBS-SSS was significantly decreased in TCBT and WCBT at 12 months follow-up. There was a greater decrease in telephone base. There was also a significant decrease in the work and social adjustment scale in both CBT groups. There was a greater decrease in web-based but not significant.	Both telephone and web-delivered CBT lead to significant improvement and life balance compared to treatment as usual in refractory IBS.
11	Berry et al., 2023 [17]	USA	Experiment Study (n = 362)	GDH resulted in significant improvement in IBS-SSS, QOL, abdominal pain, and symptom frequency. There were also improvements in stool consistency and stool frequency in all IBS subtypes.	GDH significantly improves all colon aspects of IBS and may be fruitful in controlling refractory IBS.
12	Bremner, 2013 [18]	UK	Experimental study (n = 268)	Nurse-led hypnotherapy effectively improved symptom control, QOL, and general health measures in over 80% of participants with intractable IBS.	Hypnotherapy significantly improves IBS and its associated symptoms
13	Lindfors et al., 2013 [19]	Sweden	Randomized Clinical Trial (n = 83)	After GDH, 36% of subjects were satisfied with the treatment, and 69% of subjects scored a 4 or 5 for patient satisfaction for better QOL and improvement in IBS-SSS post-treatment. Patient satisfaction was only associated with QOL domain sexual relations post-treatment.	In GDH, patient satisfaction in regard to quality of life and gastrointestinal symptoms increased.
14	Moser et al., 2013 [20]	Austria	Randomized Control Trial (n = 164)	Post-treatment, 60.8% of subjects undergoing GDH and 40.9% of supportive talks with medical treatment improved over 15 months. GDH, along with supportive talks with medical treatment, improved psychological and physical well-being incredibly, which is greater than solely supportive talks with medical treatment.	IBS-related QOL is improved by GDH and is better than only supportive talks with medical treatment. Long-term effects were seen in refractory IBS as well.
15	Vasant et al., 2020 [21]	UK	Prospective Cohort Study (n = 32)	There was a noteworthy decrease in the mean IBS-SSS following the GDH treatment compared to the initial baseline measurement.	GDH is a highly efficacious treatment for severe IBS in children and adolescents.
16	Peter et al., 2018 [22]	Austria	Experimental Study (n = 74)	In a hypnotherapy study, greater resilience, lower IBS-SSS, lower psychological distress, and QOL were found post-treatment in most subjects.	The reduction of maladaptive behaviors corresponds to resilience after hypnotherapy with a further increase in quality of life and decreases in symptoms severity and psychological distress.
17	Hasan et al., 2019 [23]	UK	Experimental Study (n = 20)	65% of participants achieved a clinically significant reduction of 50 points or more in their IBS-SSS following Skype-based gut-focused hypnotherapy. Other significant improvements included the IBS noncolonic symptom score, QOL score, anxiety score, and a nearly significant decrease in depression score.	The study demonstrated the effectiveness of Skype-based gut-focused hypnotherapy for IBS, with significant improvements in symptom severity, QOL, anxiety, and other measures as well as high quality.
18	Noble et al., 2022 [24]	UK	Prospective Cohort Study (n = 52)	52% of subjects preferred to remove over face-to-face GDH via Skype, 58% of subjects reported a 30% increase in symptom improvement, and 46% of subjects reported a greater than 30% decrease in pain reduction. 90% of subjects would suggest removing GDH to others, while 39% felt in person would be better.	GDH over Skype improved symptoms and decreased pain in subjects. The majority would recommend online GDH to themselves and others.
19	Dobbin et al., 2013 [25]	UK	Experimental Study (n = 61)	Hypnotherapy and biofeedback were equally effective in the improvement of IBS-SSS scores, anxiety, depression, and total non-gastrointestinal symptom scores.	There was no difference in success between biofeedback and hypnotherapy in the treatment of refractory IBS.
20	Gillanders et al., 2017 [26]	UK	Experimental Study (n = 24)	There were enhancements in symptom severity, GI-specific anxiety, and willingness to accept and manage IBS. However, there were no notable changes in increasing activity levels or reducing avoidance behaviors related to IBS. The intervention did not decrease the impact of IBS on participants' quality of life.	ACT-based bibliotherapy for IBS did not show a quantitative impact on IBS symptoms but should be thoroughly researched.
21	Berens et al., 2018 [27]	Germany	Randomized Control Trial (n = 30)	The results did not demonstrate significant differences between the group receiving the intervention and the control group. However, the effect size calculation showed a moderate reduction in IBS-SSS among those who underwent the group therapy.	In summary, the study found that the intervention and control groups improved IBS-SSS severity over time.

Discussion

Cognitive Behavioral Therapy

The use of CBT in refractory IBS across several studies yielded multifaceted insights into its efficacy. CBT has been shown to be efficacious for treatment-sensitive IBS, and the theory is that its success continues with refractory cases. All studies found that CBT had significantly more responders and improved the IBS-SSS and frequency of symptoms [7-14]. Lackner et al. found that CBT significantly improved cognitive flexibility from baseline compared to subjects who underwent educational training [9]. They also reported a significant improvement in the IBS-QOL in patients undergoing CBT [9]. There were significantly more responders in CBT with interoceptive exposure (CBT-IE) compared to the waitlist group, with a significant increase in IBS-SSS and IBS-QOL [8]. This improvement continued throughout the study, with few reported adverse events [8]. This emphasizes that traditional CBT is useful in treating refractory IBS with a reduction in IBS-SSS and IBS-QOL, but the mechanisms still are unclear.

Multiple studies have evaluated different delivery modes of CBT, including Lackner et al. [13], who conducted multiple secondary analyses based on standard contact S-CBT and MC-CBT. They highlighted various respondent groups with distinct responses to CBT, with significant improvements in symptom severity observed across all conditions [13]. MC-CBT patients had significantly more responders and a reduction of IBS-SSS by more than 50 points compared to S-CBT [12]. Lackner et al. identified critical mediators - IBS self-efficacy and taste agreement - linking them to treatment outcomes, emphasizing their role in treatment response and effectiveness. They found that IBS self-efficacy significantly improved in MC [11]. The average cognitive global impression (CGI) and physician-reported CGI also significantly improved in the MC-CBT group [12]. However, there was no difference between the levels of trait anxiety and anxiety sensitivity between CBT and the educational groups [10]. Satisfaction was substantial in both CBT groups compared to educational support, with MC-CBT confirming equivalence to S-CBT [12]. Ideally, MC-CBT would be easier to apply for more patients due to the ease of the delivery mode, making it superior to S-CBT.

Studies diving deep into MC-CBT compared TCBT and WCBT and found that an average of 71.9% of patients receiving TCBT had significant improvement in their symptoms at 12 and 24 months compared to 64.1% who received WCBT and 45.2% who received treatment as usual (TAU) [15,16]. The studies observed significant decreases in IBS-SSS with both, but TCBT had a greater impact with up to 40.5 point decrease [7,14-16]. There was a 6.1 increase in global improvement of symptoms (GIS) in patients receiving TCBT, compared to 3.6 in patients receiving WCBT [15]. Both CBT modes showed marked reductions in hospital anxiety and depression scores, underscoring their impact on mental health markers [15]. Both CBT groups showed significant decreases in the mean work and social adjustment scale (WSAS) scores compared to TAU [16]. There was also a significant improvement in IBS-QOL in patients undergoing both types of CBT, which further encouraged patients to continue CBT, but this was more sustained in TCBT [7]. Hughes et al. also reported a significantly high level of patient satisfaction regarding CBT treatment for their IBS [7]. While sustained improvements were evident in the CBT groups compared to TAU at the 24-month follow-up, there were some reductions in gains observed at the 12-month mark [16]. Overall, TCBT proves to be more efficacious at treating refractory IBS as a form of MC-CBT compared to WCBT; however, it appears that WCBT may be more cost-effective and easier to implement, which would lead to wide use [14].

Hypnotherapy

Studies exploring hypnotherapy's efficacy in addressing refractory IBS have revealed substantial benefits across various dimensions. In comparing GDH and TAU, there was a significant improvement in stool consistency, frequency, IBS-SSS, visual analog scale (VAS), and abdominal pain in patients who underwent GDH, as well as a decrease or cessation of medication use in most patients [17-22]. Patients undergoing GDH showed significant improvement in the IBS impact scale and in QOL [18-21]. Along with the improvement of QOL, there was a significant improvement in social function, mental health, and physical functions, which continued throughout the study [20]. Patients also had a reduced anxiety level maintained by the 10th session, further improving QOL [19,20]. However, Lindfors et al. observed that the sense of coherence and depression remained the same following treatment [19]. Satisfaction remained high throughout the treatment period in most patients using GDH [19,22]. Two studies evaluated remote GDH and observed that improvement in IBS-SSS, extracolonic symptom score, QOL, and anxiety score was still noted [23,24]. In addition, most patients were exceedingly satisfied using remote GDH [22-24]. Through GDH, patients can slow their thought processes in the moment of symptom appearance and coax themselves into an improved state. Still, this mechanism is not fully understood; studies show an association.

Of note is a study comparing biofeedback therapy for refractory IBS compared to hypnotherapy. Multiple studies did not thoroughly investigate biofeedback therapy for refractory IBS; however, Dobbin et al. evaluated its effectiveness, and the therapy was comprised of several key sessions. The initial session emphasized diaphragmatic breathing techniques, employing ECG analysis to monitor and refine breathing patterns [25]. Patients engaged in altering their breathing while receiving visual and auditory feedback on their breathing patterns and were assigned homework such as practicing 10 minutes of breathing exercises twice daily using a CD to achieve optimal resting cardiac vagal and sympathetic tone. The subsequent session replicated the breathing exercises while introducing an external stressor to evaluate stress responses and subsequent recovery [25]. Patients learned strategies to recognize and manage these internal stressors to exert control over their autonomic responses. This treatment was compared to hypnotherapy, and following the sessions, it was noted that there was a significant decrease in IBS-SSS, anxiety, depression, and extracolonic symptoms in both groups; however, there was no significant difference between them [25].

Acceptance and Commitment Therapy

While not thoroughly investigated, ACT presents an alternative paradigm for addressing refractory IBS. While CBT and GDH for IBS focus on regaining control over symptoms, ACT challenges this approach by emphasizing collaboration between therapists and clients to establish overarching life goals. Gillanders et al. implemented self-help techniques in a specialized motility clinic, providing participants with the "Better Living with IBS" book and accompanying audio exercises to address stress, symptoms, and control strategies [26]. Participants displayed enhancements in GI-specific anxiety, severity of IBS symptoms, and acceptance of IBS through ACT [26]. However, the IBS-QOL did not demonstrate significant changes in participants' QOL [26]. Notably, the IBS willingness scale exhibited notable changes, indicating a shift in acceptance, while activity engagement remained unchanged [26]. Surprisingly, there were no discernible alterations in activity engagement or unhelpful behavioral responses to IBS despite initial expectations for change with ACT therapy, highlighting nuanced outcomes in specific facets of IBS management [26].

Integrative Group Therapy

Recently, there has been an attempt to compose an integrative group therapy session that encompasses elements of psychodynamic therapy, GDH, and CBT. This innovative approach centered on the brain-gut axis as a bio-psycho-social explanatory model and was conducted by a medical specialist and psychologist [27]. The therapy spanned distinct phases, focusing on group formation, symptom understanding, coping strategies, and self-efficacy improvement. Despite significant improvements in IBS symptom severity in the intervention group, reaching a substantial reduction in symptoms with a large effect size, the comparison between the intervention and wait-listed control groups did not yield statistically significant differences [27]. While both groups showed improvements over time, the lack of statistical significance in the group differences suggests that integrative group therapy, may not be as useful in treating refractory IBS [27].

This review has limitations, centering around the lack of studies and the reduced sample size in most publications. The lack of studies investigating the different psychotherapies explained limits the ability to derive a conclusive analysis of successful therapeutic treatments. While there is a decent amount of studies evaluating the use of CBT for refractory IBS, it is inappropriate to consider it as the optimal psychotherapeutic technique as others have not been well investigated. Regarding CBT, most publications were performed by Lackner et al. and were based on a secondary analysis of their previous study. This leads to an increase in bias in the outcome of CBT and creates an overemphasis on success as they are almost equivalent to duplication of results. To balance this point, this review only focused on specific parts of each study that were not covered in the others. Lastly, because during the years of the studies, the additional therapies mentioned were relatively novel implantation, the sample sizes were small. This does the impact of the results, so caution should be taken in interpreting the results expressed.

## Conclusions

IBS is a chronic condition that hinders QOL. First-line treatment of IBS is typically through pharmacological management of core and secondary symptoms. However, in a decent amount of patients, their symptoms of IBS are not well or even managed through pharmaceutical therapy. Psychotherapy, such as CBT, GDH, biofeedback, ACT, and integrative group therapy, has been evaluated to determine if refractory cases can be managed with it. Studies concerning CBT observed significant improvement in IBS-SSS, QOL, and anxiety through the use of CBT, with WCBT showing more improvement than TCBT. WCBT was also found to be cost-effective. GDH was also significantly effective in managing IBS, and in comparison to biofeedback, both resulted in the same symptom improvement and efficacy. ACT, which uses self-help techniques, was also evaluated in one study, and there was no change in QOL; however, symptoms such as severity of IBS and anxiety did improve. Integrative group therapy combines several forms of psychotherapy previously described, but despite theories of maximal effectiveness, it shows no substantial difference in management.

IBS is a difficult condition to manage throughout life and becomes even more difficult to manage when medications become ineffective. The constant and inconvenient symptoms of abdominal pain, bloating, severe constipation, and/or severe diarrhea are a substantial burden that can lead to significant deterioration in mental health that can lead to more extracolonic symptoms. Because IBS is so common in the general population, it is imperative to identify alternative treatment modalities that further improve the symptoms associated with it, especially when pharmaceuticals fail. This review highlights the theory that psychotherapy has been researched and found to improve symptoms. Still, there is a wide variety of psychotherapy methods, which has hindered forming the ideation of one being superior. Future research should focus on the common modalities and compare these techniques to further improve refractory cases of IBS.
